# Positive Effects of Mindfulness-Based Training on Energy Maintenance and the EEG Correlates of Sustained Attention in a Cohort of Nurses

**DOI:** 10.3389/fnhum.2018.00080

**Published:** 2018-03-01

**Authors:** Kian F. Wong, James Teng, Michael W. L. Chee, Kinjal Doshi, Julian Lim

**Affiliations:** ^1^Center for Cognitive Neuroscience, Neurosciences and Behavioral Disorders Department, Duke-NUS Medical School, Singapore, Singapore; ^2^Department of Neurology, Singapore General Hospital, Singapore, Singapore

**Keywords:** mindfulness-based training, sustained attention, psychomotor vigilance test, P300, event-related desynchronization, nurses

## Abstract

Mindfulness based training (MBT) is becoming increasingly popular as a means to improve general wellbeing through developing enhanced control over metacognitive processes. In this preliminary study, we tested a cohort of 36 nurses (mean age = 30.3, SD = 8.52; 2 male) who participated in an 8-week MBT intervention to examine the improvements in sustained attention and its energetic costs that may result from MBT. Changes in sustained attention were measured using the psychomotor vigilance task (PVT) and electroencephalography (EEG) was collected both during PVT performance, and during a brief period of meditation. As there was substantial variability in training attendance, this variable was used a covariate in all analyses. Following the MBT program, we observed changes in alpha power across all scalp regions during meditation that were correlated with attendance. Similarly, PVT performance worsened over the 8-week period, but that this decline was mitigated by good attendance on the MBT program. The subjective energy depletion due to PVT performance (measured using self-report on Likert-type scales) was also less in regular attendees. Finally, changes in known EEG markers of attention during PVT performance (P300 and alpha-band event-related desynchronization) paralleled these behavioral shifts. Taken together, our data suggest that sustained attention and its associated costs may be negatively affected over time in the nursing profession, but that regular attendance of MBT may help to attenuate these effects. However, as this study contained no control condition, we cannot rule out that other factors (e.g., motivation, placebo effects) may also account for our findings.

## Introduction

Sustained attention describes the ability to focus on task-relevant behavior and resist distractibility, particularly over long periods of time. Despite the importance of sustaining attention in many on-the-job situations, attentional failures in such settings are common due to factors such as mind-wandering, motivational decline and fatigue, which can in turn lead to serious accidents (Dinges, 1995). The medical profession is a textbook example of this: impairments in sustained attention in medical settings can result in errors that may seriously compromise the safety and well being of patients. For instance, a review of the different errors made by nurses found that lack of attentiveness was an important contributor to mistakes such as missing predictable complications and inadequate monitoring of patients, with potentially catastrophic results (Benner et al., 2002). Given its importance, we conducted a study to assess whether sustained attention in nurses might be improved via mindfulness training, an intervention that may have associated cognitive benefits (Chiesa et al., 2011).

### Mindfulness Training and Sustained Attention

Traditional countermeasures to prevent dangerous lapses in attention include taking rest breaks (Tucker et al., 2003) or using stimulants such as caffeine (Bonnet et al., 1995). However, both of these are imperfect and short-term solutions. Mindfulness-based training (MBT), which focuses on developing non-judging meta-awareness of present-moment experience, (Kabat-Zinn et al., 1985) represents a promising alternative for mitigating declines in sustained attention (Chiesa and Serretti, 2010). MBT seems especially appealing as a means of stabilizing attention, as one of the skills explicitly taught in this program is how to return attention to a particular locus following the observation that the mind has wandered. Furthermore, heightened mindfulness cultivates higher levels of meta-awareness over time (Jankowski and Holas, 2014), which should in turn also lead to enhancements in sustained attention.

Despite these intuitions, studies of the effects of mindfulness on sustained attention have revealed inconsistent results, with some positive (Valentine and Sweet, 1999; Josefsson and Broberg, 2011; Jha et al., 2015) and some null findings (Tang et al., 2007; MacCoon et al., 2014). These conflicting results motivated us to conduct the current experiment using the psychomotor vigilance test (PVT; Dinges and Powell, 1985), which has unique advantages as an assay of sustained attention. In particular, successful performance of this test does not heavily require other facets of attention (e.g., orienting and/or executive attention) as compared with paradigms such as the continuous performance task (CPT; MacCoon et al., 2014) or the Sustained Attention to Response Task (SART; Jha et al., 2015). Furthermore, an earlier study showed that PVT performance improved in novice meditators following 40 min of meditation, compared to 40 min of nap or eyes opened rest (Kaul et al., 2010). Finally, the PVT is highly valid in predicting real-world performance and is already used to assess the level of impairment faced by individuals under conditions of fatigue (Lim and Dinges, 2008) and during important operations (Dinges, 2016).

The most sensitive summary metrics of PVT performance are response speed (reciprocal reaction time, or 1/RT), and the number of lapses, or reaction times over 500 ms (Basner and Dinges, 2011). Additionally, reaction times typically increase over the course of a PVT bout, a phenomenon known as the time-on-task effect (Parasuraman and Jiang, 2012). This effect is attributed to the withdrawal of attention, due in some measure to resource limits, but also to effort reallocation (Thomson et al., 2015).

In addition to measuring PVT performance, we also considered the subjective effort expended by participants using Likert-type scales assessing energy and mood. Paying attention to a task for a prolonged period of time is effortful and has a subjective energetic cost (Warm et al., 2008), exacerbating the difficulty in situations where such a lengthy period of stable attention is needed.

### Electrophysiological Correlates of Attention and Meditation

Changes in sustained attention are detectable both in behavioral and electroencephalographic (EEG) data collected during PVT performance. Objective neurophysiological markers of change are favored in mindfulness research, as they are generally less susceptible to experimenter effects and other biases. In the current study, we used two established EEG markers of attention to assess the endogenous effects of mindfulness training. First, the P3 event related potential (ERP) is often associated with directed attention (Polich and Kok, 1995), and declines in P3 amplitude reflect a reduction in the ability to allocate attention to on-task behavior due to time-on-task (Möckel et al., 2015). In vigilance paradigms, P3 amplitude is significantly greater for detected targets than omission errors (Davies and Parasuraman, 1977). Second, alpha band activity is suggested to reflect background neural noise which blocks cognitive processing (Klimesch, 1999), and individuals who display reduce alpha synchronization following a critical event show better processing of that stimulus (Yordanova et al., 2001).

Both alpha and theta power during meditation increase following training, with theta band power changes observed in individuals highly experienced in meditation (Lomas et al., 2015). In the present study, the effectiveness of 8 weeks of meditation training should be reflected in the change in electrophysiological power during meditation.

### Cognitive Benefits of Mindfulness for Nurses

Attentiveness is the key to good nursing practice (Benner et al., 2002) as nurses have to monitor therapies and patient responses over long periods and across multiple patients. Failures in monitoring may lead to both errors of omission and commission in medical practice. This becomes more prominent when nurses become tired or distracted, which are two of the three most reported causes of medical errors (Mayo and Duncan, 2004). Furthermore, lapses of attention are the most common reason for mistakes in drug administration (Härkänen et al., 2013). We believe that MBT training for nurses could lead to improvements in sustained attention, which may subsequently translate into improved patient outcomes. At the same time, we note that MBT can be challenging for some participants to adhere to, and compliance with the program may be particularly difficult in workplace settings (Van Dongen et al., 2016).

Building on the observations above, the present study examines the effects of an 8-week MBT course on PVT performance, EEG activity, and subjective energy maintenance in a group of nurses from a local public hospital. We hypothesized that PVT reaction times would improve following the intervention, that these changes would also be reflected in P3 amplitude to PVT targets, and that subjective energy expenditure on the task would decrease. Additionally, we hypothesized that these changes would be moderated by the amount of meditation training received.

## Materials and Methods

**Figure 1:**
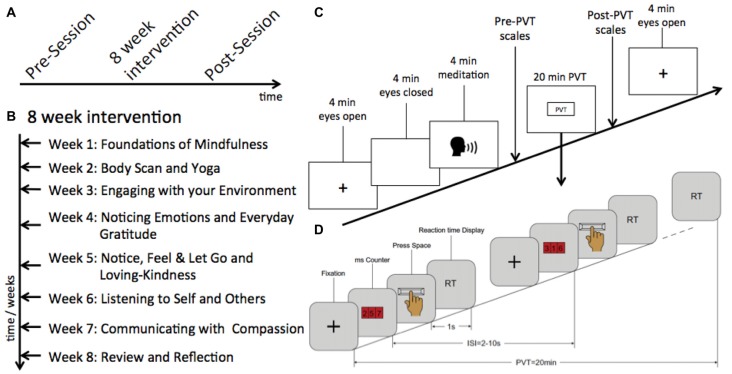
Schematics of data collection and the mindfulness-based training (MBT) program. **(A)** Sequence of sessions attended by participants. **(B)** The theme of each of the intervention sessions. **(C)** Sequence of blocks of electroencephalography (EEG) data collection across sessions. **(D)** Schematic of the psychomotor vigilance task (PVT) task.

Forty-six nurses from a local government hospital were recruited to participate in this study. Of these, 10 were excluded from the analysis as they did not return for the post-intervention data collection. The remaining 36 nurses (mean age = 30.3, SD = 8.52; 2 male) provided behavioral and EEG data in both experimental sessions, which occurred before (S1) and after (S2) a MBT program (Figure 1A). The nurses had been employed in the profession for an average of 8.52 years, with a skew towards junior nurses: 24 of the participants had been in their position for fewer than 5 years. Data were collected at the same time of day at each session for each participant to control for circadian effects. All of the nurses were shift workers, but testing sessions never occurred directly after completion of a shift. Data collection took about 2 h to complete per session and participants were compensated $40 for their time for each session. The study was approved by the Singhealth Centralized Institutional Review Board and was conducted in accordance with the ethical standards of the 1964 Helsinki declaration and its later amendments. All participants provided written informed consent.

### Mindfulness Intervention

Eight weekly 90-min sessions of MBT were conducted by an experienced mindfulness instructor (Figure 1B). These sessions were based on the mindfulness-based stress reduction (MBSR) program developed by Kabat-Zinn et al. (1985) and consisted of instruction in mindfulness practices, group sharing, and in-class activities. Both formal (e.g., mindful breathing, body scan) and informal (communication with compassion) exercises were taught as part of this program. Participants were strongly encouraged to practice the techniques taught in class for at least 15 min a day at home.

The key differences between the intervention used here and manualized MBSR were: (1) shorter session duration (1.5 vs. 2.5 h); (2) shorter duration of practice (15–20 min/day vs. 45 min per day); and (3) our intervention did not contain a retreat.

### Behavioral and EEG Data Collection

Sixty-four-channel EEG data using the standard 10–20 electrode positioning system were collected using a BrainProducts MR+ amplifier with an actiCAP at 250 Hz sampling rate, referenced at FCz and ground at Fpz. All electrode impedances were brought below 10 kΩ before the start of the recording. Five blocks of data were collected (Figure 1C): 4 min of eyes-open task-free activity, 4 min of eyes-closed task-free activity, 4 min of eyes-closed meditation with auditory instruction, a 20-min PVT (Figure 1D; Dinges and Powell, 1985; Dinges and Kribbs, 1991), and a second 4 min of eyes-open task-free activity. During the task-free (resting state) scans, participants were asked to maintain their gaze on a fixation cross on the computer monitor, and not to think of anything in particular. Subjective behavioral scales measuring energy and mood (Lim et al., 2012) were administered before and after the PVT. Additionally, sleep and wake times for the past 2 days before each recording session were collected.

### Psychomotor Vigilance Task

The PVT is a demanding test of sustained attention that is predictive of attentional lapses in operational settings (Dorrian et al., 2005; Lim and Dinges, 2010). During the PVT, participants monitor a fixed point on a display for the appearance of a millisecond counter. They are instructed to respond as quickly as possible to this counter without anticipating its appearance, following which their response time remains on screen for 1000 ms. Participants responded using the spacebar on a standard QWERTY keyboard with their dominant hand. A 20-min version of the PVT was administered to increase between-subject variance in fatigue and time-on-task (Lim et al., 2010). All stimuli were programed and presented using Psychtoolbox (Brainard, 1997).

### Behavioral Analysis

Due to the wide range of MBT sessions attended (0–8), this variable was used as a covariate in all subsequent analyses. Median reaction speed (RS), and lapses (responses >500 ms) were calculated for each participant and values for each session were entered into a one-way repeated measures ANCOVA. Subjective energy and mood scores were entered into a 2-way repeated measures ANCOVA with time (pre and post PVT) and session as factors. We correlated attendance (number of sessions) with the change in performance (response speed and lapse count) from pre- to post-intervention. We also performed correlations of attendance with the subjective measures of energy and mood.

Reported sleep times were rounded to the closest quarter hour and average sleep duration for the two nights before the EEG session was calculated. A paired sampled *t*-test was used to compare sleep duration before the pre-intervention session and before the post-intervention session.

### EEG Data Processing and Analysis

EEG data were processed in EEGLAB (Delorme and Makeig, 2004). Data were re-referenced offline to the average of mastoids ((A1 + A2)/2) and band pass filtered at 1–30 Hz. Infomax ICA was used to identify and remove eye movement components from the data. The individual alpha frequency (IAF; Klimesch, 1999) of each participant was identified and the individual alpha band was defined as the bandwidth 2 Hz above and 2 Hz below the IAF. Individual theta band was defined as the range from 4 Hz to 6 Hz below the IAF (Klimesch, 1999).

**Figure 2:**
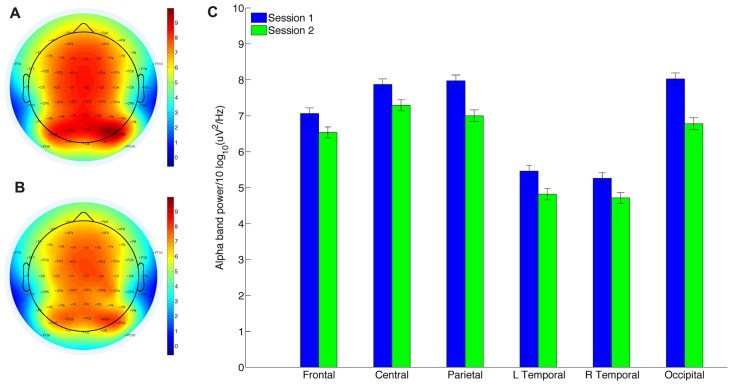
Alpha power decreases significantly post-intervention in all six recording clusters during meditation (Table 1). Topographic plot of alpha power during meditation. **(A)** Before intervention. **(B)** After intervention. **(C)** Mean alpha power for each region.

To assess whether MBT had an effect on brain activity when participants entered a mindful state, the power spectrum of the EEG data during meditation was calculated using the EEGLAB “spectopo” function (512 point FFT with 50% overlap) across six scalp regions (Table 1). This function uses Welch’s method to estimate spectral density (Welch, 1967). The change of the mean power in the individual alpha band (Figure 2; Table 2) and the individual theta band before and after intervention at each scalp region was tested using repeated measures ANCOVA.

**Table 1:** Electrodes in each region.

Region/cluster	Electrodes
Frontal	Fp1 Fp2 AF3 AF4 Fz
Central	FC1 FC2 C3 C4 Cz
Parietal	CP1 CP2 P3 P4 Pz
Left temporal	F3 F7 FC5 T7 CP5 P7
Right temporal	F4 F8 FC6 T8 CP6 P8
Occipital	PO3 PO4 O1 O2 Oz

**Table 2:** Results comparing individual alpha power during 4 min of guided meditation before and after mindfulness-based training (MBT) with FDR adjusted Q values.

	Frontal	Central	Parietal	Left temporal	Right temporal	Occipital
*F*_(1,34)_ (Q value)	14.716 (0.0008)**	17.224 (0.0001)**	18.620 (0.00002)**	13.988 (0.001)**	17.176 (0.0001)**	17.977 (0.00005)**
Correlation with attendance (Q value)	0.494 (0.001)**	0.519 (0.0002)**	0.456 (0.003)*	0.462 (0.004)**	0.524 (0.0003)**	0.436 (0.008)**
Effect Size *η*^2^	0.302	0.336	0.354	0.291	0.336	0.346

**Significant at 0.01 level; *significant at 0.05 level.

P3 (mean amplitude over 300–400 ms) ERPs were calculated using a −200 ms to stimuli onset baseline at channel Pz. Epochs were automatically flagged for removal if amplitude exceeded 3 standard deviations from the mean; visual inspection for noise was a secondary measure used prior to removal of these trials. Thirty-four of 4322 trials (0.79%) were rejected. Event related desynchronization (ERD; Pfurtscheller and Lopes da Silva, 1999), defined as mean percentage synchronization change 200–500 ms post trial onset using individual alpha bands was calculated at Pz. Mean ERP and ERD amplitude were computed for the first and last 4 min of the PVT to assess the effect of time-on-task (referred to in the ANCOVAs as “time”); we analyzed the data this way to minimize the confounding effects of between-session shifts in mean ERP/ER amplitude. Both ERP and ERD data were entered into a 2 × 2 time-by-session ANCOVA. ERP and ERD changes over time across sessions were also correlated to the number of sessions attended.

All dependent variables were evaluated for normality using Shapiro-Wilk: P3 ERP amplitudes were not normally distributed and a non-parametric (Spearman) test was used when correlating this variable.

### Subjective Scales

Subjective sensations were measured using 9-point Likert-type scales asking for ratings of sleepiness, fatigue, energy, motivation, depression, anxiety and stress; factor analysis has previously shown these to load onto two subscales: energy and mood (Lim et al., 2012). Measures from before and after PVT performance in S1 and S2 were collected and entered into a 2 × 2 repeated-measures ANCOVA.

## Results

### Training Attendance

Although the importance of regular attendance was stressed to study participants, the number of sessions attended overall was relatively low (mean (SD) = 4.14 (2.33); range = 0–8). The most common reason for missing a training session was conflict with a work shift, as these tend to be unpredictable in the population under study. To account for the large variance in sessions attended, we included this variable as a covariate in the analysis of our behavioral and EEG data, and report statistics for one-way repeated measures ANCOVA rather than main effects.

### Sleep Duration

Self reported sleep durations were collected for the two nights prior to each EEG testing session. We found no differences between the averaged sleep duration per night prior to the two recording sessions (S1 (SD): 431.5 min (71.2); S2: 440.6 min (78.0), *t* = −0.538, *p* = 0.594, *d* = −0.0922).

### EEG Spectral Analysis of Meditation

We assessed whether the EEG power spectrum of participants differed when they were instructed to enter a state of mindfulness after the 8-week period of training. To test this, we compared both alpha and theta power over six scalp regions during meditation in S1 and S2. Alpha power changed significantly across all regions (Figure 2) after correction for multiple comparisons and the degree of change was significantly correlated with MBT attendance over all regions (Table 2). Although there was a group decrease in alpha power across sessions, examination of individual differences showed that participants with higher attendance tended to have relatively stable or increased levels of alpha in S2 (Table 2). Theta power changes from S1 to S2 were not significant across any region, and were not correlated with MBT attendance.

### Psychomotor Vigilance Test Performance

To assess performance on the PVT, we extracted two variables from each 20-min test bout: median response speed (RS; reciprocal RT, or 1/RT, with RT measured in milliseconds), and lapses. These variables are the most sensitive markers of sustained attention in this task (Basner and Dinges, 2011), and are also highly reliable over time (Dorrian et al., 2005).

Compared with previous studies of participants in a well-rested state (Lim and Dinges, 2010), we observed that average performance on the PVT was relatively poor (median (standard deviation (SD)) RS: 2.917 (0.280) ms^−1^; lapses: 12.85 (12.73)). While we would traditionally reject participants who commit at least 20 or more lapses (RT > 500 ms; Lim et al., 2012), here, this would result in the removal of 13 participants from the sample of 36.

Inspection of the data suggested that the high lapse count in this dataset was driven by generalized response slowing, and not stochastic attentional failures. As such, we used a more liberal threshold for classifying lapses, which was defined as the median + 2 SD of the RT (mean (SD) RT = 711 (62) ms) of the PVT performance in S1, calculated individually for each participant. This individual lapse threshold was applied to both S1 (mean (SD) lapse count = 7.6 (3.5)) and S2 (mean (SD) lapse count = 9.81 (8.75)) PVT results. Using this criterion, only one remaining participant committed an excessive number of lapses in S2 PVT (40), 3.5 SD. over the mean; this dataset was removed from further analysis that required lapse trials to be rejected.

**Figure 3:**
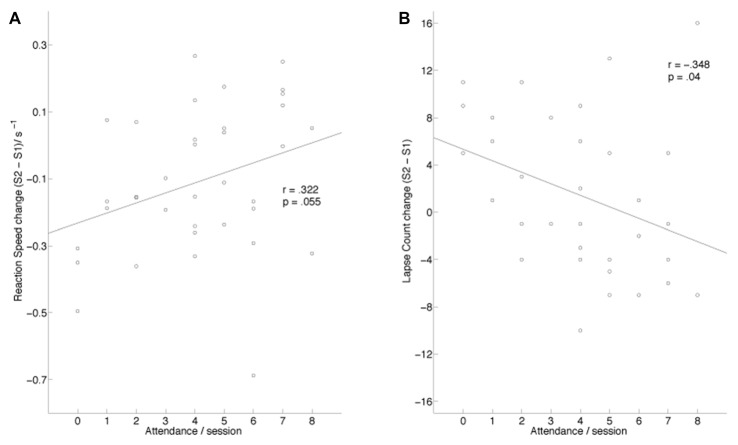
**(A)** Change in median response speed (S2–S1) is significantly correlated with MBT attendance. **(B)** Change in number of lapses (S2–S1) is significantly correlated with MBT attendance.

Using one-way ANCOVA, we found a significant effect of session on RS (*F*_(1,34)_ = 10.571, *p* = 0.003, $\eta_{\text{p}}^{2}$ = 0.237) and lapses (*F*_(1,33)_ = 6.177, *p* = 0.018, $\eta_{\text{p}}^{2}$ = 0.158). On the average, performance in S2 was worse, with slower RS (S1 median (SD) RS: 2.97 (0.29); S2 median (SD) RS: 2.86 (0.28)) and more lapses (S1 mean (SD): 7.60 (3.45); S2 mean (SD): 8.94 (7.16)). To assess the direction of effect of the covariate, we correlated attendance with change in response speed (Figure 3A) and lapses (Figure 3B) and found a marginally significant correlation with RS (*r* = 0.322, *p* = 0.055) and a significant correlation with lapses (*r* = −0.348, *p* = 0.04). Higher attendance was associated with better performance in S2 vs. S1 for both RS and lapse counts. We note from the scatter plots that this correlation was driven both by improved performance in regular attendees as well as degraded performance in individuals who attended little to none of the program.

### ERP Analysis

**Figure 4:**
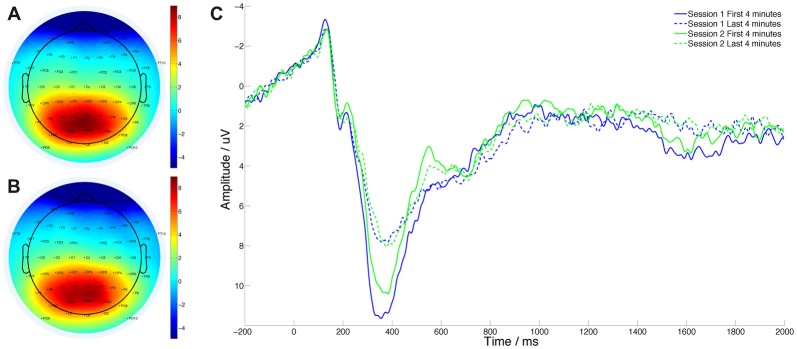
Grand average P3 EEG amplitude topographic plot during PVT. **(A)** Before intervention. **(B)** After intervention. **(C)** Grand average event related potential (ERP) at channel Pz.

P3 amplitude to correct, non-lapse responses was measured and averaged for the first and last 4 min of each 20-min PVT bout (Figure 4). Using repeated-measures ANCOVA, we found a significant effect of session (*F*_(1,33)_ = 6.829, *p* = 0.013, $\eta_{\text{p}}^{2}$ = 0.171) and time (*F*_(1,33)_ = 9.049, *p* = 0.005, $\eta_{\text{p}}^{2}$ = 0.215) on this variable. The interaction between session and time was also significant (*F*_(1,33)_ = 7.667, *p* = 0.009, $\eta_{\text{p}}^{2}$ = 0.189), driven by smaller decreases in P3 amplitude over time in the post intervention session. ERP change values over time and session were not significantly correlated with attendance (*r* = −0.259, *p* = 0.133).

### ERD Analysis

**Figure 5:**
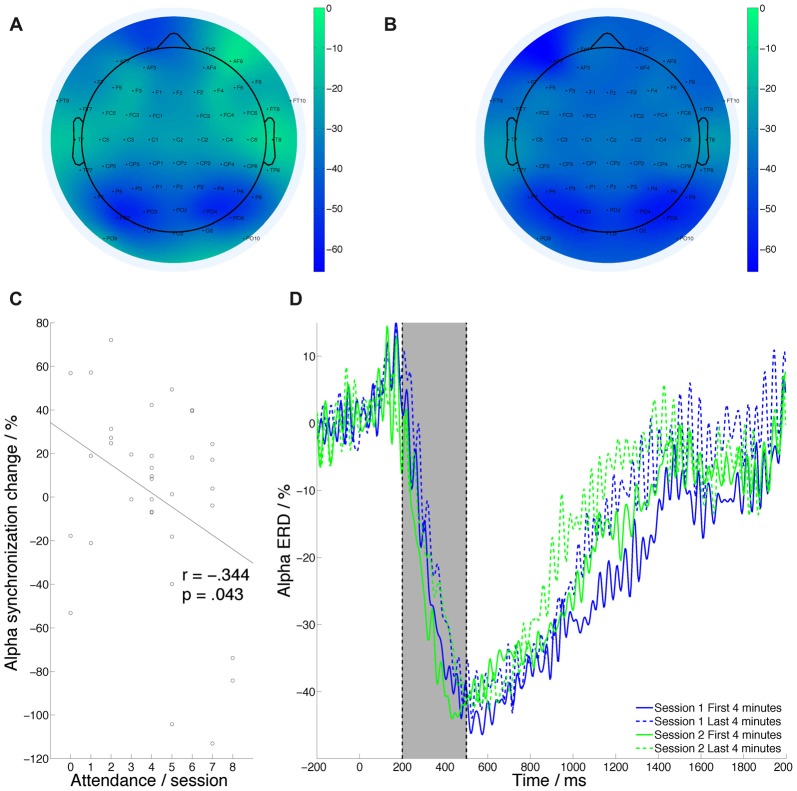
Grand average event related desynchronization (ERD) topographic plot during PVT performance. **(A)** Before intervention. **(B)** After intervention.** (C)** ERD change across task and session is significantly correlated with MBT attendance. **(D)** Grand average ERD.

The same criteria for trial averaging were used for ERDs (Figure 5) and ERPs. There was no significant effect of session on alpha ERD (*F*_(1,33)_ = 0.157, *p* = 0.694, $\eta_{\text{p}}^{2}$ = 0.005) and a trend effect of time (*F*_(1,33)_ = 3.681, *p* = 0.064, $\eta_{\text{p}}^{2}$ = 0.1). The session by time interaction was marginally significant (*F*_(1,33)_ = 3.472, *p* = 0.71, $\eta_{\text{p}}^{2}$ = 0.093).

ERD changes across time and session (i.e., the change in the time-on-task effect on ERDs) were significantly negatively correlated with number of the MBT sessions attended (Figure 5C; *r* = −0.344, *p* = 0.043), demonstrating reduced alpha power synchronization with more sessions of MBT training attended.

### Subjective Data

One participant did not provide self-report data in S2, and was excluded from the analysis. We found a significant effect of time (*F*_(1,33)_ = 5.679, *p* = 0.023, $\eta_{\text{p}}^{2}$ = 0.147) and a significant interaction between session and time (*F*_(1,33)_ = 5.034, *p* = 0.032, $\eta_{\text{p}}^{2}$ = 0.132) on subjective energy. When energy changes were correlated with attendance, we found that nurses who attended more sessions reported smaller decreases in subjective energy following PVT performance (*r* = 0.372, *p* = 0.028). No significant changes were seen in subjective mood scales.

## Discussion

In this intervention study, we investigated the effects of MBT on a widely used test of sustained attention: the Psychomotor Vigilance Test (PVT). In this experiment, we measured behavioral, subjective, and electrophysiological changes during performance of the 20-min PVT in a group of nurses before and after an 8-week MBT protocol. Attendance to the course was relatively low, and perhaps because of this, we did not find support for the hypotheses that sustained attention and its associated EEG markers would improve significantly in the whole group of trainees. However, when attendance was introduced as a covariate, our data show significant changes in PVT response speed and lapse counts, with better performance observed in nurses who attended more of the MBT. Similarly, we observed a significant session by time interaction in P3 amplitude, with high attendees showing a smaller time-on-task effect. Finally, nurses who attended more of the intervention reported lower subjective energy decreases as a result of performing the PVT. Overall, these data suggest that mindfulness training enhances the ability to sustain attention, with associated and measurable neurophysiological and subjective changes.

### MBT Has a Beneficial Effect on Sustained Attention

Evidence of enhanced sustained attention has been found in those who already have meditation experience compared to novice meditators (Valentine and Sweet, 1999; Josefsson and Broberg, 2011). However, studies examining the effects of short-term mindfulness training on sustained attention have yielded mixed results. For example, studies using the attention network test (ANT) have shown both improvements and null effects in all three (alerting, orienting, executive) attentional networks in different experimental settings (Jha et al., 2007; Tang et al., 2007). In contrast, no differences were found on CPT performance by Halperin et al. (1991) or Anderson et al. (2007) in studies comparing MBT to a waitlist control or treatment as usual. Most recently, using the SART, Jha et al. (2015) found that MBT improved accuracy (measured by A’) on this task in both a military and a civilian cohort.

The heterogeneity of study designs and outcome measures in the literature makes it challenging to draw conclusions from the currently available data. Furthermore, none of the studies above assessed sustained attention using the PVT, which is particularly useful for the assessment of operational readiness (Balkin et al., 2004). Importantly, the PVT is highly sensitive to changes to changes in alertness and vigilance due to factors such as sleep deprivation (Lim and Dinges, 2010), and shift work (Neri et al., 2002), and is suitable for measuring inter-individual differences in sustained attention across these states (Van Dongen and Dinges, 2005).

Analysis of the PVT data revealed a pattern of behavior over time whereby those with poor attendance in the MBT program tended to have relatively worse performance, while regular attendees showed a slight tendency to improve. Duration of sleep reported before each testing session did not differ significantly, suggesting that our results were not caused by differences in homeostatic sleep pressure. As the test-retest reliability of the PVT performed in a similar state is high (0.88; Dorrian et al., 2005), it is likely that the changes observed over time are due to enhancement or attenuation of the capacity to sustain attention. This improvement may be due to a number of different factors including increased awareness of mind wandering and other off-task behavior (Cheyne et al., 2009; Mrazek et al., 2013), an expanded resource pool of attentional resources to deploy towards on-task behavior, or better alertness due to improved sleep (Black et al., 2015).

We note that the overall comparison of response speed and lapses showed that performance was significantly worse in S2. This effect was driven largely by poor attendees. The data suggest that the high-stress environment in the workplace had adverse cognitive consequences on nurses’ cognitive functioning (Shapiro et al., 2005; Yang et al., 2016; Burton et al., 2017), but that mindfulness training mitigated and even reversed the negative effects of the stress. Our results echo the findings of Leonard et al. (2013), who also found declines in attention over a similar span of time in a group of incarcerated youth, with protective effects conferred by MBT.

### MBT Reduces the Energetic Cost of Sustaining Attention

In addition to the objective changes on the PVT, we observed that performing the PVT resulted in overall reductions in subjective feelings of energy (i.e., increases in sleepiness and fatigue), but that nurses with higher MBT attendance experienced a lower magnitude of decrease. Reductions in energy due to sustaining attention are commonly reported in the literature (Warm et al., 2008; Helton and Russell, 2011; Lim et al., 2012), and are thought to reflect resource expenditure due to task demands. Thus, the findings in the subjective scales support the theory that MBT induces either in greater availability or more efficient deployment of attentional resources during times of high resource demand (Thomson et al., 2015). This result may be of direct relevance to reducing the mental fatigue caused by performing nursing duties that is an indirect cause of nursing errors (Mayo and Duncan, 2004).

### MBT Enhances Alpha Power During Meditation

We measured EEG power during a 4-min period of guided mindful breathing in order to assess spectral differences associated with MBT. We focused our analysis on the alpha and theta bands of the EEG spectrum, as these bands have shown the most robust changes following such training in the prior literature (Lomas et al., 2015). Consistent with this literature, we found significant differences across regions in the alpha band, with greater alpha increases associated with higher MBT attendance. This suggests that the intervention was effective in teaching participants who attended the program how to achieve a mindful state. No differences were found in the theta band.

Changes in alpha power are putatively driven by increases in internally directed attention (Klimesch et al., 2007), corresponding with relative elevation in activity in task-negative brain regions (Knyazev et al., 2011). Thus, our results suggest that participants were better able to focus attention internally and inhibit distraction/mind-wandering during meditation following MBT, but only if they had relatively high attendance to the program.

Theta power is an index of executive function, and it has been suggested that greater theta synchronization in meditators marks the increased capacity to control one’s locus of attention (Cahn and Polich, 2006). However, in contrast with alpha, differences in theta power are not as consistently reported in experienced meditators, and indeed, we did not observe significant theta associations in the current work. This suggests that there may be factors (e.g., length of experience, type of meditative discipline) that moderate theta power differences in the mindful state after MBT.

### MBT Reduces P3 Attenuation Over Time During Task Performance

We analyzed the P3 component of responses to PVT targets and found a significant effect of the intervention when attendance was included as a covariate. Although the direct correlation did not reach significance, P3 amplitude tended to be less attenuated with time-on-task when participants attended more of the MBT program. In undemanding tasks, the P3 is thought to be an index of resource allocation with greater peak amplitude signifying greater arousal and attention (Polich, 2007). As such, our data suggest that MBT changed either the ability or the willingness of participants to deploy resources consistently over the full duration of the PVT.

Previous studies of mindfulness practitioners have been consistent in finding modulation of the P3 component in comparison with controls (for a review, see Cahn and Polich, 2006). These changes typically fall into one of three categories.

First, several studies have reported reductions in P3 amplitude to salient but task-irrelevant stimuli in mindfulness practitioners, suggesting that superior attention in this group is due to an improvement in top-down inhibition. For example, Cahn and Polich (2009) studied experienced Vipassana meditators as they performed an auditory oddball task, and found P3a amplitude differences (as well as differences in N1 and P2) to rare distracter stimuli, but not to standard stimuli or oddballs. In a similar fashion, Moore et al. (2012) found that participants in the mindfulness-training arm of a randomized control trial (vs. a waitlisted group) had reduced P3 amplitude to incongruent stimuli on a color-word Stroop test.

Second, MBT can also enhance P3 amplitude to attended and relevant stimuli. Experienced Vipassana meditators show greater P3b amplitudes to targets on an auditory oddball task after meditation (Delgado-Pastor et al., 2013). Lakey et al. (2011) showed that a brief mindfulness induction boosted P3 amplitudes and improved accuracy in a brain-computer interface task. More recently, Smart et al. (2016) demonstrated such increases in a group of patients with subjective cognitive impairment (but not healthy controls) who performed a Go/No-Go task before and after MT.

Finally, there is evidence that MT can change the way in which limited attentional resources are deployed over time. In an elegant study of the attentional blink phenomenon, Slagter et al. (2007) showed that, following a 3-month meditation retreat, P3b amplitude was reduced for initial targets (reflecting lower attentional capture), thus reducing attentional-blink size, and improving detection of the second (blink) target. These data suggest a potential unifying mechanism for the changes described in the prior two paragraphs—meditation-trained participants might improve the ability both to upregulate resources devoted to relevant stimuli and downregulate resources devoted to distracting stimuli based on overall resource reallocation.

As the PVT only has a single stimulus type (in contrast to Go/No-Go or oddball tasks), we cannot make nuanced inferences about the functional significance of the P3 in this task. Nevertheless, our data are broadly compatible with the theory that MBT results in a more optimal allocation of resources to task-relevant behavior (Davies and Parasuraman, 1977). Additionally, our results suggest that these resources are more evenly deployed over time, mitigating the traditional declines in P3 amplitude that are seen with time-on-task (Koelega et al., 1992; Möckel et al., 2015; Staub et al., 2015).

### MBT Stabilizes Alpha ERD Over Time During Task Performance

The final noteworthy result observed in this study was a significant reduction in the change in alpha-band ERD to target stimuli across the PVT from S1 to S2. Desynchronization in this band to targets reflects suppression of background noise and devotion of processing resources to the relevant stimulus (Pfurtscheller and Lopes da Silva, 1999). As with P3 amplitude, we observed a significant effect of time on ERD, suggesting that participants found it increasingly difficult to effectively allocate attentional resources to the task. Following MBT, those with good attendance were less vulnerable to this effect over time, possibly because of an improved ability to suppress background neural noise.

### Limitations

The primary limitation of the current study is the lack of a control group, or randomization to condition, the gold standard of any intervention study being a double-blind randomized controlled trial. Due to logistical constraints, we were not able to conduct this form of trial in this study. As our design uses comparisons of pre- and post-intervention outcomes, we cannot make strong causal claims about whether MBT *per se* led to our observed changes. Nevertheless, one merit of the current design is that it is a good reflection of the likely results of a MBT course being offered in the real world.

Second, it may be possible that differences in motivation might account for the results. Individuals participating in the program who are more highly motivated would likely attend more of the training may put in more effort while performing the PVT whilst participants less invested in the program may attend less or none of the training and put minimal effort into PVT performance. To address this point, we compared motivation across sessions using one of the items administered on the pre-task questionnaire (“How motivated are you”). Using a repeated measures ANCOVA with attendance as a covariate, we found no significant difference on this item (*F*_(1,34)_ = 3.356, *p* = 0.76, *η*^2^ = 0.09), indicating that motivation was unlikely to account for the other observed changes.

Third, attendance to our program was relatively poor, limiting our ability to study the effect of a full MBT course on changes in cognition. Other investigators have also reported that it can be challenging to get participant compliance with mindfulness training in workplace settings (Van Dongen et al., 2016). Nevertheless, as a consequence of this spread in attendance, we were able to provide evidence that sustained attention might get worse over time in a stressful working environment, and that it may be important for those undergoing mindfulness training to attend a majority of the training sessions in order to reap its benefits on attention.

## Conclusion

In summary, our data support the hypothesis that attending MBT is an effective means of mitigating or reversing the negative effects of work stress on sustained attention. However, we caution that less weight should be placed on the evidence presented here in comparison with other studies using stronger designs, particularly randomized controlled trials.

Attention is a prerequisite for higher cognitive operations, and failures of sustained attention or vigilance can lead to serious errors, particularly in medical practice (Barger et al., 2006; Scott et al., 2006). Here, we show a tripartite benefit of MBT on this domain, with a protective effect on behavior, electrophysiology, and subjective energy expenditure. MBT is beginning to show promise as a low-cost, easily disseminable means of combating failures of sustained attention, and further study is warranted to reveal the active ingredients of this intervention and the best ways in which it may be delivered.

## Author Contributions

The study design was concieved by JL, KD and MWLC. Stimuli was prepared by KFW and KD. Data was collected by JT and KFW. Data analysis was performed by KFW. JL, KFW and MWLC contributed to writing of the manuscript.

## Conflict of Interest Statement

The authors declare that the research was conducted in the absence of any commercial or financial relationships that could be construed as a potential conflict of interest.
